# Altered Mental Status in an Octogenarian: How Frequently Should Serotonin Syndrome Be Considered?

**DOI:** 10.7759/cureus.57403

**Published:** 2024-04-01

**Authors:** Saliha Erdem, Ahmad Damlakhy, Kristin Konja, Sifullah Bashar

**Affiliations:** 1 Internal Medicine, Wayne State University School of Medicine, Detroit, USA; 2 Internal Medicine, Detroit Medical Center, Sinai Grace Hospital, Wayne State University, Detroit, USA; 3 Internal Medicine, Detroit Medical Center, Michigan State University, Detroit, USA

**Keywords:** chronic kidney insufficiency, opioid use, serotonin norepinephrine reuptake inhibitor, altered mental state, serotonin syndrome (ss)

## Abstract

Serotonin toxicity, an adverse consequence of elevated serotonin levels in the brain, poses a considerable threat to life. Antidepressants, frequently prescribed for various conditions in older adults, such as depression, anxiety, and sleep disturbances, significantly contribute to this risk. The elderly are particularly vulnerable due to multiple comorbidities, cognitive decline, medication interactions, polypharmacy, and chronic kidney disease. This case underscores the critical importance of considering serotonin syndrome as a potential diagnosis in patients using serotonin and norepinephrine reuptake inhibitors, especially within vulnerable populations. Here, we present the case of an 89-year-old female who presented with altered mental status and a hypertensive emergency. Following a thorough examination and exclusion of alternative causes of acute encephalopathy, serotonin syndrome induced by the use of venlafaxine and oxycodone was diagnosed.

## Introduction

Serotonin syndrome (SS) represents a life-threatening complication arising from substances known to elevate serotonin levels [1]. It most commonly occurs due to drug interactions involving the concurrent administration or overdose of two or more agents that enhance serotonergic neurotransmission through diverse mechanisms [2]. Patients typically manifest a constellation of neuromuscular, autonomic, and mental status symptoms. This condition often arises from the concomitant use of medications such as selective serotonin reuptake inhibitors (SSRIs), serotonin-norepinephrine reuptake inhibitors (SNRIs), monoamine oxidase inhibitors (MAOIs), and tricyclic antidepressants (TCAs). Additionally, illicit substances like amphetamines, opioids, and cocaine may precipitate serotonin syndrome. Venlafaxine, a serotonin-norepinephrine reuptake inhibitor (SNRI), has been identified among the medications capable of precipitating serotonin syndrome [3]. With the escalating use of antidepressants in the United States over recent decades, serotonin syndrome has become an increasingly significant clinical concern. However, given that serotonin syndrome is a diagnosis of exclusion and its symptomatology spans a broad spectrum, prompt diagnosis and initiation of therapy are imperative in clinical practice [3,4].

## Case presentation

An 89-year-old female with a medical history including a right lung lower lobe adenocarcinoma, resected 12 years ago, hypertension, hyperlipidemia, type 2 diabetes mellitus, chronic obstructive pulmonary disease on 4L home oxygen, chronic kidney disease (CKD, stage III), and heart failure with preserved ejection fraction, presented to the emergency department with altered mental status. According to family members, she was typically awake, alert, and oriented, using a walker for ambulation and requiring assistance with dressing and bathing. Prior to hospital admission, she complained of epigastric pain without associated nausea, vomiting, or diarrhea. However, her mental status deteriorated gradually, rendering her nonverbal and prompting the family to seek medical attention. Her social history was negative for alcohol, tobacco, and illicit drug use. Home medications are outlined in Table 1.

**Table 1:** Demonstrating the list of the patient's home medications mg - milligrams; BID - twice daily; QHS - every night; mL - milliliter; mg/mL - milligrams per milliliter; PRN - as needed

Medication	Dosage	Frequency
Bumetanide	2 mg	BID
Aspirin	81 mg	Daily
Atorvastatin	40 mg	QHS
Clopidogrel	75 mg	Daily
Doxazosin	1 mg	Daily
Nifedipine	30 mg	Daily
Spiriva	1 puff	Daily
NovoLog	7 units	3 times/day
Lantus	17 units	Daily
Albuterol-ipratropium	3 mL	PRN
Oxycodone	5 mg	PRN
Venlafaxine	37.5 mg	Daily
Acetaminophen-hydrocodone	500 mg	Daily
Losartan	50 mg	BID
Megestrol	40 mg/mL	Daily
Memantine	10 mg	QHS
Triamcinolone topical	0.10%	PRN

Upon presentation, the patient was awake but demonstrated a lack of alertness or orientation and was unable to follow commands, albeit maintaining airway protection. She exhibited severe distress and agitation, along with normal conjunctiva, mydriatic pupils, and ocular myoclonus. Palpation revealed diffuse abdominal pain without rebound or guarding. Neurological examination revealed generalized rigidity with muscle hypertonicity, accompanied by a tremor in the bilateral upper extremities, but no neck stiffness or photophobia. Evaluation of deep tendon reflexes was hindered by excessive muscle rigidity and shivering. The patient's skin appeared warm, moist, flushed, and diaphoretic.

**Figure 1:**
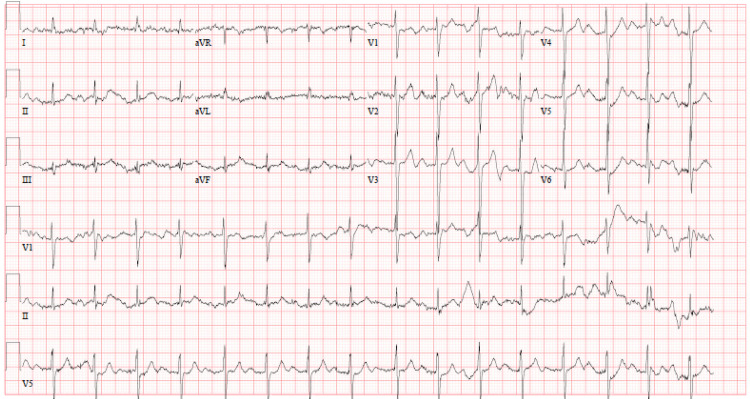
12 lead electrocardiogram on admission showing sinus rhythm with a heart rate of 98 beats per minute and a first-degree atrioventricular block

She was hypertensive (blood pressure 242/106 mmHg), tachycardic (heart rate of 108 beats per minute), tachypneic (respiratory rate of 36 breaths per minute), and had an oxygen saturation of 98% on nasal cannula with 4L of oxygen, and fever with a rectal temperature of 39°C. A 12-lead electrocardiogram (EKG) revealed sinus rhythm with a heart rate of 98 beats per minute and a first-degree atrioventricular block, exhibiting a normal axis with no significant ST segment or T-wave abnormalities (Figure 1). 

**Figure 2:**
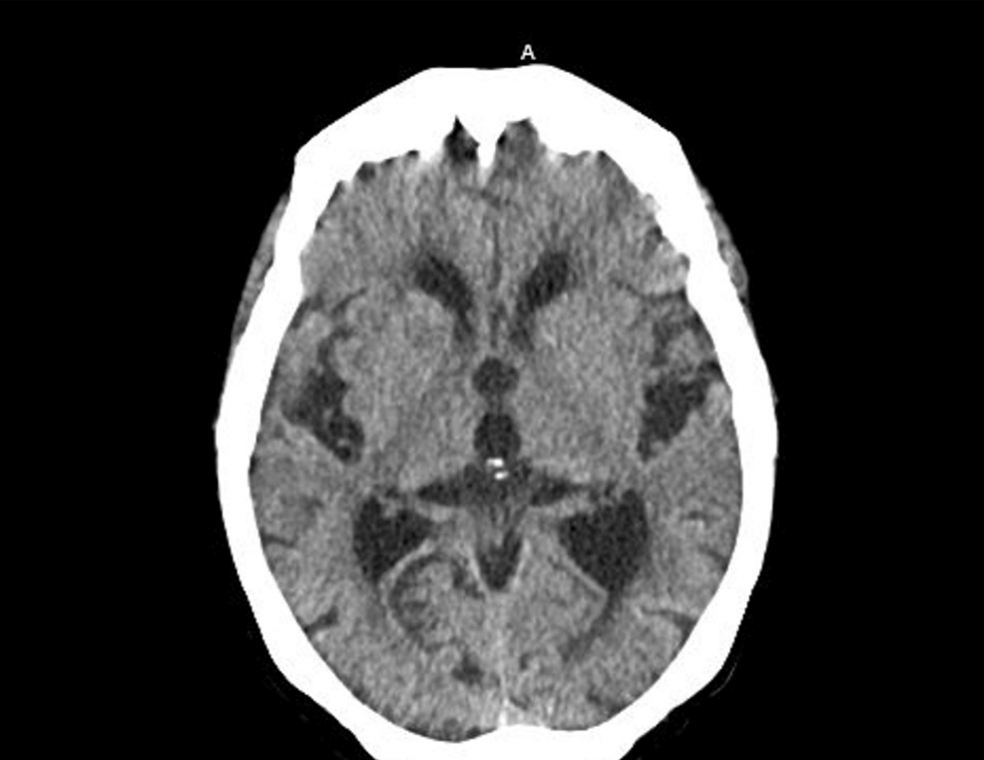
Computed tomography on admission showing no acute intracranial process with progression of diffuse cerebral atrophy, chronic microangiopathic ischemic changes, and an old lacunar infarct in the right basal ganglia

Computed tomography (CT) of the head demonstrated no acute intracranial process but displayed progression of diffuse cerebral atrophy (Figure 2).

**Figure 3:**
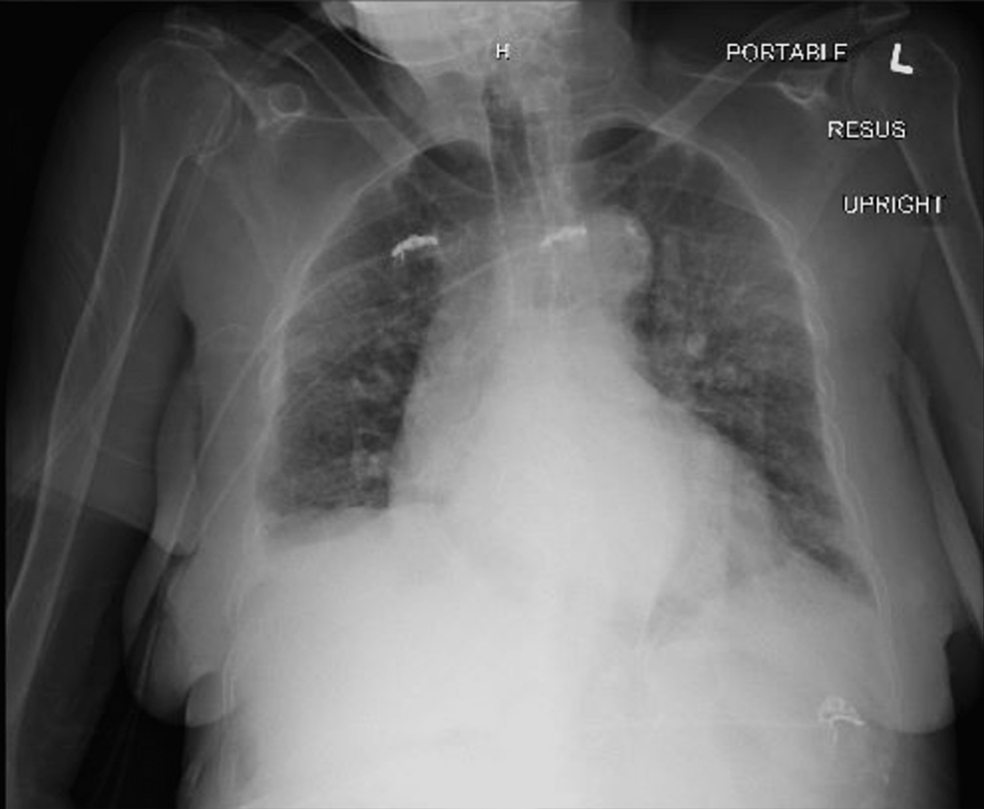
Chest X-ray on admission showing left perihilar hazy, ill-defined opacity with previous right lower lobe lobectomy

Due to suspicion of sepsis, a chest X-ray was obtained and revealed a left perihilar hazy, ill-defined opacity, noting the previous right lower lobe lobectomy (Figure 3). 

Urinalysis with microscopic examination did not reveal any urinary tract infection, while a urine drug screen tested positive for opiates. Respiratory panel testing excluded influenza A, B, respiratory syncytial virus (RSV), or severe acute respiratory syndrome coronavirus 2 (SARS-CoV-2). Blood workup indicated leukocytosis, hypokalemia, and elevated liver function enzymes, as illustrated in Table 2.

**Table 2:** Blood workup result mMol/L - millimoles per liter; mg/dL - milligrams per deciliter; mL/min/1.73 m2 - milliliters per minute per 1.73 square meters; mm3 - cubic millimeter; mmHg - millimeters of mercury; mEq/L - milliequivalents per liter; GFR - glomerular filtration rate

Laboratory Test	Admission value	Normal ranges
Lactic acid (mMol/L)	1.4	0.4-2.0
Lipase (Units/Liter)	15	11-82
Sodium (mMol/L)	144	136-145
Potassium (mMol/L)	3.2	3.5-5.1
Chloride (mMol/L)	103	98-107
Carbon dioxide (mMol/L)	31	21-31
Anion gap (mMol/L)	10	5-15
Aspartate aminotransferase (Units/Liter)	178	13-39
Alanine aminotransferase (Units/Liter)	97	7-52
Glucose (mg/dL)	164	75-105
Urea nitrogen (mg/dL)	28	7-25
Creatinine (mg/dL)	1.18	0.60-1.20
Calcium (mg/dL)	9.4	8.6-10.8
Magnesium (mg/dL)	1.8	1.6-3.0
Phosphorus (mg/dL)	2.6	2.5-4.5
GFR (mL/min/1.73 m2)	52	60-120
White blood cells (mm3)	24.2 k	4,500-11,000
Arterial blood gas pH	7.474	7.35-7.45
Arterial blood gas PCO2 (mmHg)	35.6	36-45
Arterial blood gas PO2 (mmHg)	69.9	80-100
Arterial blood gas HCO3 (mEq/L)	25.7	22-28
Arterial blood gas O2 saturation (percent)	92.9	96-98

Given the patient's fever, leukocytosis, and chest X-ray findings suggestive of pneumonia, she was initiated on ceftriaxone and doxycycline for suspected community-acquired pneumonia. Blood cultures were obtained, revealing the presence of *Streptococcus gallolyticus* (susceptible to ceftriaxone). Additionally, due to the patient's abdominal pain and signs of sepsis, a contrast-enhanced CT scan of the abdomen and pelvis was conducted, which did not reveal any acute abdominal abnormalities. Despite 72 hours of antibiotic therapy and supportive measures, the patient's mental status failed to improve, and she remained hypertensive and agitated, with no alleviation of rigidity, ocular myoclonus, or fever.

Consequently, considering the diagnostic exclusion and the patient's physical examination findings, serotonin syndrome was diagnosed, prompting consultation with the medical intensive care unit (ICU) on hospital day three. Throughout this period, the patient maintained airway protection despite altered mentation and did not require intubation. Blood pressure was managed with hydralazine and nitroglycerin, and agitation was addressed with diazepam. Subsequently, cyproheptadine was administered. Following the implementation of supportive measures and cyproheptadine therapy, the patient's encephalopathy, physical examination findings, and vital signs demonstrated improvement. She remained in the medical ICU for four days before being successfully transferred to the medical floor for further management. The patient's discharge occurred on hospital day nine. Her mentation continued to improve, along with successful blood pressure control.

## Discussion

Serotonin syndrome (SS) is a rare and potentially life-threatening condition resulting from an excessive accumulation of serotonin, which leads to the overactivation of central and peripheral postsynaptic 5HT (5-hydroxytryptamine)-1A and 5HT-2A receptors [1]. A range of medications, including opioids, amphetamines, antidepressants, antiemetics, antihistamines, and illicit substances such as cocaine and 3,4 methylenedioxymethamphetamine (MDMA or "ecstasy"), are recognized culprits in precipitating SS [1]. This syndrome manifests as a triad of symptoms comprising altered mental status, neuromuscular abnormalities, and autonomic hyperactivity, which may present as hyperreflexia, myoclonus, rigidity, restlessness, delirium, confusion, diaphoresis, hypertension, hyperthermia, mydriasis, and tachycardia [5].

In our case, the patient was concurrently using oxycodone, an opioid, and venlafaxine, a serotonin-norepinephrine reuptake inhibitor (SNRI). Both medications possess serotonin reuptake inhibitor characteristics, and their combination is believed to have precipitated SS in this instance. Furthermore, depression is prevalent among individuals with chronic medical conditions, resulting in increased antidepressant use and potentially higher rates of SS in the elderly population [3]. Our patient, who had multiple comorbid chronic conditions, was utilizing venlafaxine for depression and oxycodone for pain relief.

The diagnosis of SS is predominantly clinical and is one of exclusion. It's noteworthy that our patient underwent a comprehensive workup, including head CT imaging to rule out cerebrovascular events, assessment for electrolyte and metabolic imbalances, and urinalysis for drug screening. Although her urine drug screen was positive only for opioids, there were no signs of cardiorespiratory depression or other physical exam findings indicative of possible opioid intoxication. Additionally, she received appropriate treatment for community-acquired pneumonia (CAP) based on culture results, yet her mental status did not improve despite antibiotic therapy for 48 to 72 hours. Moreover, she remained hypertensive, tachycardic, and agitated despite antihypertensive medications and supportive measures. Given the lack of improvement in her mental status, along with persistent autonomic abnormalities and neuromuscular hyperactivity, the diagnosis of SS was made. The patient's favorable response to benzodiazepine and cyproheptadine further supported this diagnosis.

Early recognition and prompt treatment of SS are crucial to prevent morbidity and mortality. Treatment typically involves discontinuing all serotonergic medications and providing supportive care to maintain stable vital signs. Mild cases may require observation, supportive care, and, if necessary, benzodiazepines for sedation. Moderate cases may benefit from cyproheptadine administration, although the evidence supporting this intervention is limited. Severe cases, characterized by life-threatening hyperthermia, necessitate urgent intensive care management. Generally, the prognosis for SS is favorable when diagnosed and treated promptly. In cases of uncertainty regarding diagnosis, discontinuation of serotonergic agents and initiation of supportive care are recommended [5,6].

In our patient, management involved the use of nitroglycerin and hydralazine to control hypertension, diazepam to mitigate adrenergic symptoms, and cyproheptadine. Notably, opioids, when combined with antidepressants, may elevate the risk of precipitating SS [7]. Opioids inhibit the serotonin transporter, increasing serotonin concentration in the synaptic cleft, and are also believed to enhance intrasynaptic serotonin release [1,8]. Combined with SNRIs, this interaction may result in serotonin toxicity and the development of SS, as observed in our patient who was using both oxycodone and venlafaxine. The elderly population, with age-related changes affecting drug metabolism, is particularly vulnerable to medication accumulation. Venlafaxine and oxycodone, metabolized primarily in the liver by CYP2D6/3A3/4 isoenzymes, may pose an increased risk of SS in individuals with genetic polymorphisms [5]. Additionally, age-related declines in liver function and renal clearance, particularly in patients with underlying chronic kidney disease (CKD), may exacerbate medication accumulation and predispose this population to medication-related side effects, including SS [9].

Despite the potential severity of SS, its true incidence remains unaccounted for [5]. Some cases may go undiagnosed due to milder symptoms, limited exposure to the condition, lack of awareness, and variable diagnostic criteria. Given the diverse array of medications capable of inducing SS and the overlap in symptoms with other conditions, clinicians must consider a broad differential diagnosis, including malignant hyperthermia, neuroleptic malignant syndrome, anticholinergic toxicity, drug intoxication, encephalitis, heat stroke, and serotonergic discontinuation syndrome [3]. Increasing clinician awareness of drug interactions, medication accumulation, and metabolic activity is crucial in preventing SS. Patients prescribed medications with serotonergic effects should be educated about the early signs and symptoms of SS. Clinicians should exercise caution when prescribing serotonergic medications to the elderly, regularly review medication regimens, and prioritize patient safety. Education of both clinicians and patients may aid in reducing the prevalence of SS and enhancing the management of individuals presenting with its signs and symptoms [10].

## Conclusions

Serotonin syndrome (SS) represents a significant clinical challenge due to its potentially life-threatening nature and diverse etiologies, ranging from medication interactions to age-related changes in drug metabolism. Our case highlights the importance of recognizing SS promptly, particularly in vulnerable populations such as the elderly with multiple comorbidities. Early diagnosis is crucial, necessitating a comprehensive clinical evaluation and exclusion of alternative diagnoses. Treatment strategies should focus on discontinuing serotonergic medications and providing supportive care to stabilize vital signs, with consideration given to the severity of symptoms. Clinician awareness of drug interactions, medication accumulation, and metabolic activity is paramount in preventing SS and optimizing patient outcomes.
